# Effect of Methyl Bromide Additions on the Flame Speed of Methane

**DOI:** 10.6028/jres.067A.009

**Published:** 1963-02-01

**Authors:** Carl Halpern

## Abstract

The effect of small quantities of methyl bromide, up to 0.5 percent by volume, on the flame speed of methane-air mixtures has been determined. Maximum flame speeds, at given experimental conditions, are reduced proportionately to the amount of methyl bromide added. Flame speeds of rich mixtures are reduced much more than are flame speeds of lean mixtures. Reaction zone thickness of methane-air flames is increased by the addition of methyl bromide and the thickness increases with the amount of methyl bromide.

## 1. Introduction

Halogenated hydrocarbons have long been used to extinguish fires, and the effect of these combustion inhibitors on various combustion parameters has been the subject of many investigations [1–10].^1^ Limits of flammability, quenching distance, and laminar flame speed all have been found to be affected by these inhibitors. Effect of inhibitors on the limit of flammability has been the chief topic of these investigations. The effect on flame speed has been little studied and what work that has been published usually treats of the effects of several inhibitors on a given fuel; description of the effects of other experimental conditions are usually lacking [1, 2, 9, 10]. It was felt that a study of the effect of one inhibitor on flame speed under controlled experimental conditions would be of interest and of value. Methane was chosen as the fuel since we have had considerable experience in the determination of the flame speed of methane-air mixtures. Methyl bromide was chosen as the inhibitor.

## 2. Apparatus and Procedure

A description of the apparatus and method used to measure flame speeds has been presented earlier [14]. Briefly, the apparatus consists of drying and metering systems for air and fuel, and a nozzle, the exit of which is the burner port. Means are provided to control the temperature of the combustible mixture issuing from the nozzle. Flame speeds are determined by a total-area method, which is based on the measurement of the area of an enlarged photograph of the schlieren image of the flame.

It was decided to prepare mixtures of air and methyl bromide of the desired strength and to meter these mixtures, rather than to set up a third metering system for the small quantities of methyl bromide that would be required. Some error is introduced in the metering of these mixtures, since the sharp-edged orifice used was calibrated for air. However, since the maximum amount of methyl bromide added was only 0.5 percent by volume of the air, it was considered that the error would be tolerable.

The mixtures of methyl bromide and air were prepared in a 120 gallon (16 ft^3^) steel tank. The tank was evacuated to a pressure of several microns and the methyl bromide admitted from its container. Rise in pressure was measured on a mercury manometer, read to 0.01 in. Air from a compressor was dried by passing first through a column of activated alumina and then through a cold trap immersed in a slush of dry ice in a mixture of equal parts by weight of chloroform and carbon tetrachloride. Water content of the air is thus kept at 0.03 percent by volume. The dried air was admitted slowly to the tank, and the final pressure, generally 130 psig, was read on a calibrated Bourdon gage to 0.1 psig.

The products of combustion, which contain hydrogen bromide and bromine, were drawn from the enclosure surrounding the burner by a large capacity vacuum pump. Air currents, set up by the pump, so disturbed the flames that the pump was shut off when photographs were taken.

Mixtures of 0.1, 0.2, 0.3, 0.4, and 0.5 percent by volume of methyl bromide in air were prepared. For each mixture, the variation of flame speed with mixture ratio, by weight, of methane to air plus methyl bromide was determined, gas velocity at the exit of the nozzle being constant. The ratio, by weight, of methane to air plus methyl bromide was varied from 0.054 to 0.072, and the gas velocity at the port of the nozzle was varied from 3 to 6 fps. Control of the temperature of the combustible mixture was such that the maximum change in temperature during a single run of about 3 hr duration was 3.7 °F. Actual gas temperatures ranged from about 90 °F in the summer to about 75 °F in the winter. The variation of flame speed with temperature was determined using a mixture ratio of methane to air of 0.060 (the air contained 0.2% methyl bromide), and a gas velocity of 4 fps; the temperature range in this determination was from 70 to 95 °F. Variation of flame speed with temperature was found to be 0.00328 fps per °F, and this value was used to correct all flame speeds to a constant temperature of 75 °F. It is not expected that the rate of change of flame speed with temperature will differ appreciably with the relatively small amounts of methyl bromide added. Some previously unpublished data in our possession shows that the rate of change of flame speed with temperature is unaffected by changes in mixture ratio.

## 3. Results

Combustion of methane with air to which methyl bromide had been added proceeded smoothly. The outer mantle of the flames was colored brown by the formation of free bromine and the odor of bromine was noticeable. At lean conditions, the brown color began close to the base of the flame and extended to the tip. As flames became richer in fuel, the normal blue-green color of the inner cone appeared and the brown color was seen only near the tip of the outer mantle. As the concentration of methyl bromide was increased, the brown color became more intense, but in rich flames was visible only near the tip of the outer mantle. It is probable that hydrogen bromide is the original product and is converted to bromine by the overall reaction: 4 HBr+O_2_—— — → 2 Br_2_+2 H_2_O. Apparently there is insufficient oxygen in a rich flame for complete conversion of hydrogen bromide.

**Figure 1:**
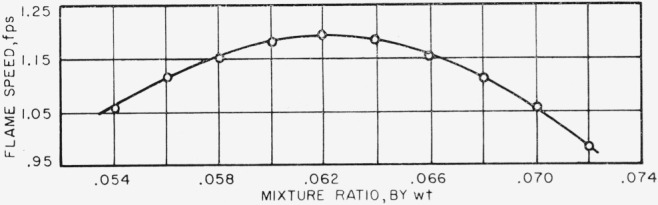
Variation of flame speed of methane with mixture ratio.

The variation of flame speed with mixture ratio for a methane-air flame is shown in figure 1. This is taken from our previous work [14] with the values of flame speed corrected to 75 °F. The maximum flame speed is 1.196 fps, at a fuel-air ratio of 0.062. At a fuel-air ratio of 0.054, flame speed is 1.057 fps, and at a fuel-air ratio of 0.072, flame speed is 0.980 fps.

**Figure 2:**
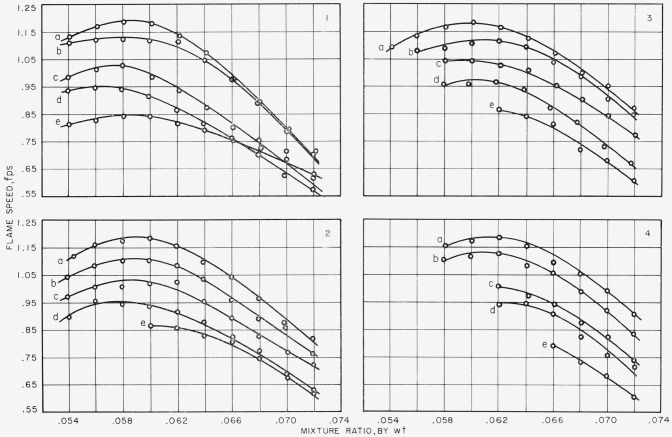
Effect of addition of methyl bromide on the flame speed of methane. Percentage methyl bromide added: *a*=0.1; *b*=0.2; *c*=0.3; *d*=0.4; and *e*=0.5. Gas velocity=1, 3 fps; 2, 4 fps; 3, 5 fps; and 4, 6 fps.

Results of addition of methyl bromide are shown in figures 2 through 6. It should be noticed that gas mixtures described as having the same mixture ratio but with different amounts of methyl bromide added do not have exactly the same ratio of fuel to air. As the amount of additive increases, the amount of air decreases, and the ratio between fuel and air increases; but this increase is only a matter of about a tenth of a percent. However, these facts should be remembered when comparing results. Figure 2 shows the variation of flame speed with mixture ratio, by weight, of methane to air plus methyl bromide, when 0.1, 0.2, 0.3, 0.4, and 0.5 percent by volume, respectively, of methyl bromide is added to air, and at gas velocities of 3, 4, 5, and 6 fps.

For each addition of methyl bromide and at each gas velocity, there is found one value of mixture ratio at which flame speed is greatest. Maximum flame speed for a methane-air flame occurs at mixture ratio 0.062; addition of methyl bromide causes maximum flame speed to shift to leaner conditions, especially at the lower gas velocities. (Stoichiometric for a methane-air flame is equivalent to a mixture ratio of 0.0583.) Table 1 lists the maximum flame speeds and the experimental conditions at which they were observed.

Flash-back which represents the rich limit of operation of the burner was not encountered at any of the experimental conditions used. Blow-off which represents the lean limit of operation was rather frequent especially at gas velocities of 5 and 6 fps. No cases of blow-off were encountered at 3 fps and only one at 4 fps; this occurred at mixture ratio of 0.058 with 0.5 percent CH_3_Br added to the air. Table 2 lists the conditions at which the leanest flame could exist before blow-off occurred. It can be noted that as the amount of methyl bromide increases, the lean limit shifts toward fuel-rich conditions, while the flame speed becomes less. Rich flames are thus stabilized when methyl bromide is present.

Theoretically, the velocity with which combustible gas issues from a burner should have no effect on the flame speed. In practice, some variation of flame speed with gas velocity is noted [14]. In this present work, a small decrease of flame speed with increasing gas velocity, amounting to about 2 percent of the average value over the gas velocity range covered, was found at mixture ratios yielding maximum flame speeds, and at leaner conditions. At rich conditions, flame speed increased with gas velocity, and the variation amounted to as much as 15 percent of the average value. Rich flames, however, are very tall as the flame speed is very low, and tall flames are very susceptible to disturbances. It is possible, therefore, that the increased variation of flame speed with gas velocity may be due to these disturbances.

The addition of methyl bromide to the combustion air reduces the maximum flame speed and the more methyl bromide added, the greater is the reduction in flame speed. Flames burning at mixture ratios greater than that at which maximum flame speed occurred are more affected by methyl bromide than are those burning at leaner conditions. At 0.1 percent methyl bromide addition, for example, and at a gas velocity of 3 fps, the maximum flame speed is 1.186 fps at a mixture ratio of 0.058. At a mixture ratio of 0.072 flame speed is 0.718 fps which is 60.6 percent of the maximum, while at mixture ratio 0.054, flame speed is 1.134 fps which is 95.6 percent of the maximum. The corresponding percentages for the methane-air flame are 82.0 percent at mixture ratio 0.072 and 88.4 percent at mixture ratio 0.054. The addition of 0.1 and 0.2 percent of methyl bromide even increases the flame speed at lean conditions. At a gas velocity of 3 fps, the flame speed at mixture ratio 0.054 is 1.134 fps for a 1 percent addition and 1.120 fps for 0.2 percent addition. For a methane-air flame, flame speed is 1.057 fps at mixture ratio 0.054 and a gas velocity of 6 fps.

**Figure 3:**
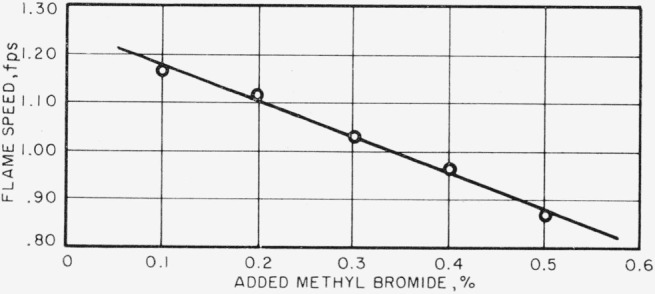
Variation of flame speed with added methy bromide. Mixture ratio, by weight, =0.062. Gas velocity=5 fps.

**Figure 4:**
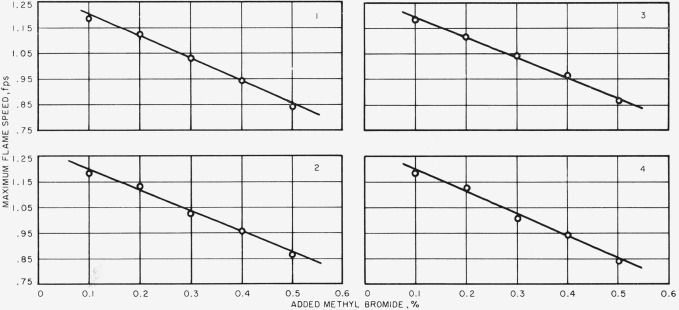
Variation of maximum flame speed with added methyl bromide. Gas velocity—1, 3 fps; 2,4 fps; 3, 5 fps: and 4, 6 fps.

In figure 3, flame speed is plotted against the percent of methyl bromide added to the combustion air. Mixture ratio is 0.062 and gas velocity is 5 fps. Flame speed decreases as the percentage of methyl bromide increases, and the data points fall along a straight line. For all experimental conditions of mixture ratio, gas velocity and methyl bromide addition, similar results were found. Hence, it may be deduced that the reduction in flame speed is directly proportional to the amount of methyl bromide, at least in the range of addition used in this work. In figure 4, the maximum flame speed at each addition of methyl bromide, at constant gas velocity, is plotted against the percentage of methyl bromide, and again the data points fall on a straight line, and the slopes of these lines are of similar magnitude. The reduction in maximum flame speed is directly proportional to the amount of methyl bromide added and amounts to 0.0828 fps for each tenth percent of methyl bromide added.

In the method used for determining flame speeds, photographs of the schlieren and visible images of the flame are taken simultaneously on the same film. The schlieren image which depends on the change in density and thus on the change in temperature marks the position where chemical reactions begin in the flame [15]. The visible image indicates the region in the flame where reactions are completed [16] except for equilibrium changes. Hence, the separation between the schlieren and visible images is a measure of the thickness of the reaction zone. However, since both images in an enlarged photograph are rather diffuse, these measurements cannot be considered exact.

**Figure 5:**
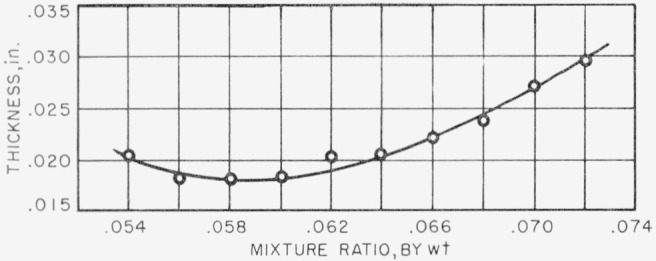
Variation of reaction zone thickness with mixture ratio. 0.2 percent methyl bromide added. Gas velocity=4 fps.

**Figure 6:**
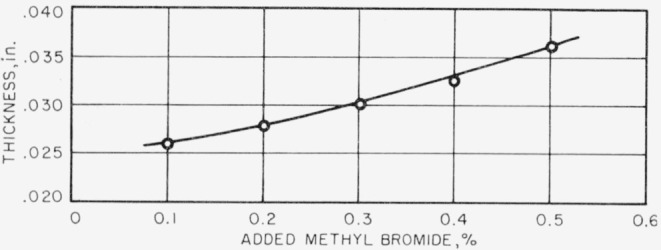
Variation of reaction zone thickness with added methyl bromide. Mixture ratio 0.070. Gas velocity=6 fps.

It is found that the thickness of the reaction zone varies with the flame speed; the greater the flame speed, the less is the thickness of the reaction zone. Figure 5 shows the variation of reaction zone thickness with mixture ratio at constant gas velocity; 0.2 percent methyl bromide was added to the air and the gas velocity was 4 fps. Similar curves are obtained for all the other experimental conditions. Figure 6 shows the variation of reaction zone thickness with added methyl bromide at constant mixture- ratio and at constant gas velocity. Mixture ratio was 0.070 and gas velocity was 6 fps. Zone thickness increases with the amount of methyl bromide added, and similar results were obtained at all other experimental conditions of mixture ratio and gas velocity. From some previously unreported experiments, it is found that the reaction zone thickness of a methane-air flame at fuel-air ratio of 0.070 and gas velocity of 6 fps was 0.0180 in. Minimum thickness was 0.015 in. and occurred at mixture ratio 0.062.

The mechanism of combustion inhibition by halogenated hydrocarbons is not fully understood [5, 9, 11, 12]. Since the combustion of hydrocarbons in air involves the propagation of chain reactions by free radicals, it is plausible to assume that the presence of halogen results in the deactivation of one or more of the chain carrying radicals. Since deactivation would effectively decrease the rate of reaction, the general effect of chemical inhibitors in decreasing flame speeds would be explained. The increased reaction zone thickness which results on the addition of methyl bromide may also be explained by the effective decrease in reaction velocity.

The increase in flame speed noted at lean conditions at additions of 0.1 and 0.2 percent methyl bromide cannot be so explained. Since there is more oxygen in a lean flame, it may be that methyl bromide acts as a fuel rather than an inhibitor at this condition and the reaction mechanism is different. If methyl bromide acts as a fuel, then the mixture ratio should be expressed as $\frac{\text{wt methane}+\text{wt methy bromide}}{\text{wt air}}$, in which case the value is 0.057 instead of 0.054, and 0.059 instead of 0.056 for the addition of 0.1 percent methyl bromide. Then if we superpose the curve for flame speed versus mixture ratio at 0.1 percent methyl bromide addition, using the new values of mixture ratio, on the curve for methane-air, the two curves agree for values of mixture ratio less than that at which maximum flame speed occurred.

## 4. Experimental Observations

Table 3 presents in detail observations on the effect of some variables on the flame speed and reaction zone thickness.

## 5. Conclusions

Small amounts of methyl bromide added to methane-air mixtures have a large effect on the flame speed. Maximum flame speed is reduced proportionately to the amount of methyl bromide added. Flame speed of rich mixtures is much more reduced than that of lean mixtures. Reaction zone thickness is increased by the presence of methyl bromide.

## Figures and Tables

**Table 1:** Maximum flame speeds

Flame speed	Wt CH_4_	Percent CH_3_Br added to air	Flame speed	Gas velocity
	Wt air+CH_3_Br		Flame speed CH_4_-air	
				
*fps*				*fps*
1.196	0.062	0	……………………	6
1.186	.058	0.1	0.992	3
1.125	.058	.2	.941	3
1.029	.058	.3	.860	3
0.945	.056	.4	.790	3
.843	.060	.5	.704	3
1.186	.060	.1	.992	4
1.103	.060	.2	.922	4
1.029	.062	.3	.860	4
0.958	.056	.4	.801	4
.864	.060	.5	.722	4
1.185	.060	.1	.991	5
1.117	.062	.2	.934	5
1.047	.058	.3	.875	5
0.967	.062	.4	.806	5
.867	.062	.5	.724	5
1.186	.062	.1	.992	6
1.127	.062	.2	.942	6
1.006	.062	.3	.841	6
0.945	.064	.4	.790	6
a.790	.066	.5	.661	6

a Blow-off occurred at 0.064.

**Table 2:** Lean limit of operation of burner

Percent CH_3_Br added to air	Wt CH_4_	Gas velocity	Flame speed
	Wt air+CH_3_Br		
			
		*fps*	*fps*
0.1	0.058	6	1.156
.2	.056	5	1.084
.2	.058	6	1.103
.3	.058	5	1.047
.3	.062	6	1.006
.4	.058	5	.958
.4	.062	6	.941
.5	.060	4	.864
.5	.062	5	.867
.5	.066	6	.790

**Table 3:** Effect of some variables on flame speed

A. 0.1% CH_3_Br added to air				
Wt CH_4_	Reaction zone thickness	Gas temperature	Flame speed	Flame speed corrected to 75° F
Wt air+CH_3_Br				
1. Gas velocity=3 fps				
	*in.*	°*F*	*fps*	*fps*
0.05407	0.0155	91.3	1.187	1.134
. 05608	.0146	91.4	1.224	1.170
. 05807	.0168	90.0	1.234	1.186
. 06013	.0165	90.6	1.236	1.185
. 06216	.0168	90.9	1.190	1.138
. 06410	.0190	91.2	1.127	1.074
. 06610	.0200	91.3	1.032	.979
. 06800	.0223	91.4	.944	.891
.07013	.0257	91.4	.834	.780
.07214	.0305	91.3	.771	.718
2. Gas velocity=4 fps				
	*in.*	°*F*	*fps*	*fps*
0.05436	0.0192	89.6	1.168	1.120
.05592	.0172	87.8	1.203	1.167
.05794	.0179	88.3	1.217	1.174
.05992	.0202	88.9	1.232	1.186
.06189	.0191	89.2	1.203	1.156
.06387	.0195	89.6	1.143	1.094
.06594	.0219	89.6	1.090	1.042
.06794	.0235	89.3	1.014	.967
.06989	.0272	89.3	.923	.876
.07196	.0295	89.3	.866	.818
3. Gas velocity=5 fps				
0.05409	0.0198	86.6	1.133	1.094
.05593	.0191	87.1	1.174	1.135
.05808	.0203	87.0	1.207	1.168
.06010	.0191	84.8	1.217	1.185
.06204	.0224	85.8	1.201	1.167
.06406	.0228	86.1	1.162	1.126
.06604	.0218	86.6	1.108	1.070
.06809	.0238	87.0	1.050	1.011
.07006	.0267	86.9	.991	.952
.07204	.0284	87.0	.904	.864
4. Gas velocity=6 fps				
0.05807	0.0190	86.1	1.192	1.156
.06003	.0203	86.2	1.208	1.171
.06200	.0192	85.6	1.223	1.186
.06402	.0212	84.9	1.186	1.153
.06601	.0225	85.4	1.129	1.095
.06800	.0238	86.0	1.092	1.056
.07003	.0260	86.1	1.027	.991
.07198	.0287	86.3	.943	.906
B. 0.2% CH_3_Br added to air				
1. Gas velocity = 3 fps				
	*in.*	°*F*	*fps*	*fps*
0.05406	0.0217	74.5	1.109	1.110
.05601	.0202	74.8	1.121	1.120
.05803	.0201	75.2	1.126	1.125
.05998	.0181	75.2	1.120	1.120
.06206	.0207	75.5	1.115	1.113
.06402	.0221	75.3	1.049	1.048
.06599	.0236	76.0	.983	.980
.06794	.0251	75.6	.890	.889
.07000	.0279	75.2	.787	.786
.07202	.0328	75.1	.706	.706
2. Gas velocity = 4 fps				
0.05394	0.0203	77.7	1.053	1.044
.05591	.0182	75.8	1.088	1.085
.05794	.0182	75.8	1.105	1.103
.05996	.0183	75.9	1.106	1.103
.06197	.0203	76.1	1.092	1.088
.06391	.0205	76.4	1.038	1.033
.06594	.0220	76.9	.966	.960
.06787	.0237	77.2	.899	.892
.06994	.0272	77.4	.859	.851
.07197	.0295	79.5	.778	.764
3. Gas velocity = 5 fps				
0.05600	0.0190	77.2	1.091	1.084
.05800	.0178	77.5	1.098	1.090
.05998	.0198	76.7	1.115	1.110
.06199	.0200	76.6	1.122	1.117
.06396	.0207	76.6	1.102	1.097
.06597	.0218	76.8	1.043	1.037
.06801	.0230	76.9	.995	.989
.06999	.0258	77.1	.912	.906
.07198	.0278	77.2	.882	.874
4. Gas velocity = 6 fps				
	*in.*	°*F*	*fps*	*ps*
0.05798	0.0195	77.8	1.112	1.103
.05994	.0212	77.8	1.126	1.117
.06199	.0207	78.7	1.139	1.127
.06397	.0207	76.2	1.087	1.083
.06597	.0230	76.7	1.064	1.058
.06804	.0247	77.2	.996	.989
.06995	.0278	77.6	.931	.922
.07197	.0295	77.8	.844	.836
C. 0.3% CH_3_Br added to air				
1. Gas velocity = 3 fps				
	*in.*	°*F*	*fps*	*fps*
0.05391	0.0237	80.6	1.004	0.985
.05602	.0253	80.0	1.029	1.013
.05799	.0220	79.6	1.044	1.029
.06019	.0234	79.8	1.001	.985
.06210	.0258	80.3	.956	.939
.06414	.0237	80.4	.892	.874
.06610	.0258	80.2	.817	.800
.06810	.0295	80.6	.747	.729
.06997	.0349	79.2	.701	.687
.07196	.0395	79.3	.631	.617
2. Gas velocity = 4 fps				
0.05390	0.0203	78.3	0.984	0.973
.05594	.0200	77.3	1.016	1.008
.05796	.0190	76.5	1.013	1.008
.05996	.0228	76.7	1.024	1.019
.06193	.0217	77.1	1.036	1.029
.06393	.0252	78.3	.965	.954
.06596	.0240	79.1	.909	.896
.06794	.0260	78.9	.839	.827
.07002	.0299	77.9	.778	.769
.07196	.0297	77.2	.729	.722
3. Gas velocity = 5 fps				
0.05804	0.0210	77.4	1.055	1.047
.06000	.0222	76.6	1.045	1.040
.06211	.0207	77.5	1.037	1.029
.06410	.0215	76.2	1.013	1.009
.06613	.0242	76.8	.959	.953
.06807	.0270	77.2	.913	.906
.07006	.0300	77.0	.847	.840
.07203	.0350	76.5	.757	.752
4. Gas velocity = 6 fps				
0.06196	0.0232	77.8	1.015	1.006
.06418	.0247	77.4	.981	.974
.06607	.0272	78.9	.955	.942
.06804	.0283	77.3	.884	.877
.07006	.0300	77.4	.833	.825
.07198	.0345	77.8	.749	.740
D. 0.4% CH_3_Br added to air				
1. Gas velocity=3 fps				
	*in.*	°*F*	*fps*	*fps*
0.05391	0.0203	72.5	0.930	0.938
.05593	.0207	73.9	.941	.945
.05795	.0209	75.0	.942	.942
.05993	.0218	75.4	.918	.917
.06194	.0225	75.8	.872	.869
.06393	.0248	74.6	.811	.812
.06596	.0270	72.7	.745	.752
.06790	.0315	72.9	.708	.701
.06988	.0372	74.6	.628	.623
.07195	.0408	75.3	.576	.575
2. Gas velocity=4 fps				
	*in.*	°*F*	*fps*	*fps*
0.05395	0.0228	74.8	0.895	0.896
.05591	.0227	75.1	.958	.958
.05790	.0205	75.2	.945	.944
.05995	.0217	74.8	.935	.936
.06193	.0237	74.7	.916	.917
.06395	.0248	74.7	.877	.878
.06600	.0280	74.7	.804	.805
.06798	.0303	74.8	.770	.771
.07000	.0360	75.2	.677	.676
.07196	.0397	75.4	.628	.627
3. Gas velocity=5 fps				
0.05795	0.0225	75.3	0.959	0.958
.05978	.0227	75.3	.957	.957
.06177	.0237	74.8	.967	.967
.06375	.0258	73.2	.934	.940
.06578	.0272	74.5	.872	.873
.06775	.0300	75.2	.822	.821
.06978	.0340	75.1	.730	.730
.07175	.0393	74.2	.672	.674
4. Gas velocity=6 fps				
0.06202	0.0233	84.6	0.972	0.941
.06399	.0248	82.8	.971	.945
.06598	.0260	83.0	.934	.908
.06801	.0297	81.0	.843	.823
.07001	.0325	81.6	.778	.757
.07204	.0380	82.9	.741	.716
E. 0.5% CH_3_Br added to air				
1. Gas velocity=3 fps				
	*in.*	°*F*	*fps*	*fps*
0.05399	0.0248	80.1	0.830	0.814
.05602	.0235	79.7	.844	.829
.05802	.0233	79.9	.858	.842
.06003	.0238	76.8	.849	.843
.06200	.0240	77.6	.826	.818
.06397	.0250	78.3	.801	.790
.06596	.0265	78.6	.773	.761
.06797	.0282	78.6	.769	.757
.07001	.0328	80.0	.734	.718
.07204	.0370	79.6	.629	.614
2. Gas velocity=4 fps				
0.06000	0.0230	78.7	0.876	0.864
.06192	.0232	79.3	.871	.856
.06396	.0252	79.7	.844	.829
.06594	.0278	80.0	.811	.794
.06793	.0307	80.5	.761	.743
.07002	.0330	80.7	.706	.688
.07194	.0387	81.0	.633	.614
3. Gas velocity=5 fps				
0.06196	0.0275	75.0	0.867	0.867
.06398	.0275	75.4	.841	.840
.06593	.0311	74.6	.813	.814
.06793	.0325	75.5	.719	.718
.06994	.0377	75.6	.682	.679
.07199	.0422	75.6	.604	.603
4. Gas velocity=6 fps				
0.06599	0.0293	76.4	0.795	0.790
.06801	.0323	75.5	.735	.733
.06999	.0362	76.0	.684	.681
.07200	.0415	75.8	.603	.601

## Footnotes

1 Figures in brackets indicate the literature references at the end of this paper.
